# *N*-Heterocyclic analogues as peptide deformylase inhibitors: Molecular modeling, synthesis and antibacterial evaluation

**DOI:** 10.1186/1471-2334-14-S3-E18

**Published:** 2014-05-27

**Authors:** Anuradha Singh, Madhu Yadav, Preeti Singh, Ritika Srivastava, Nidhi Singh, Rajesh Verma, Ramendra K Singh

**Affiliations:** 1 Nucleic Acids and Antiviral Research Laboratory, Department of Chemistry, University of Allahabad, Allahabad-211002, India

## Background

Global emergence of worsening antibiotic resistance has become a serious problem because of emergence of multi drug resistance (MDR) strains. The emergence of MDR strains has forced the scientific community to searching of new antibiotics.

## Methods

A new series of *N*- heterocyclic compounds has been derived from benzimidazole and pyrimidine nuclei optimized with the Discovery studio 3.0 software to investigate the interactions between the target compounds and the amino acid residues of *Escherichia coli* PDF Ni (PDB: ID 1G2A), and then synthesized. Further, all compounds were examined for their antibacterial activities against Gram-positive, *S.viridians*, and Gram-negative bacterial strains, *E. coli*, *P. mirabilis* and *K. pneumoniae* using the microdilution broth susceptibility test method and subjected to polynomial regression.

## Results

The compounds showed promising *in silico* results as reflected by their significant scoring functions and close interatomic contacts through strong H-bonds with Ile 44, Gly 45, Gly 89, Cys 90, Glu95, Cys129 and Arg 97; pi-pi interaction with Arg 97 and His 132. Antibacterial results indicated that these molecules possessed significant activity against all the tested species with MIC values ranging between 0.22 and 2.62 μM. The regression coefficients (R^2^ = 0.78 to 0.89) derived for each strain were correlated significantly.

## Conclusion

The molecules proved as effective inhibitors of bacterial PDF, which has been corroborated by docking studies.

